# Thermal Simulation Analysis of Internal Control Circuit Board of Steering Gear Box Based on COMSOL Three-Dimensional Simulation Software

**DOI:** 10.1155/2022/3006349

**Published:** 2022-03-24

**Authors:** Wen Huo

**Affiliations:** School of Mechano-Electronic Engineering, Xidian University of Technology, Xian 710000, China

## Abstract

The steering gear device includes two parts, a steering gear control circuit and a transmission component. The transmission component includes a ball screw and a motor. During the operation of the steering gear, due to the presence of the steering gear ball screw motor and friction, a certain amount of heat will be generated, which will affect the steering gear control circuit in a confined space. At the same time, the steering gear is inevitable in the actual working process, and will experience a high temperature environment, which will increase the temperature of the internal structure of the steering gear, and due to the difference in thermal expansion coefficients between various materials, stress and strain will occur in the structure, which may cause mismatch or even cracks in the system structure, and the steering gear system cannot work normally. It is necessary to analyze the thermal characteristics of the overall steering gear under multiple factors. Based on this, this paper uses COMSOL three-dimensional simulation software to conduct thermal simulation analysis on the shell of the steering gear containing the control circuit board. The temperature distribution and stress-strain response law of the control circuit board in the box, and the influence of different materials and thickness of the box heat insulation layer on the thermal characteristics of the control circuit are discussed, and then a reasonable thickness and material of the heat insulation layer are obtained for the design of the rudder chassis for reference.

## 1. Introduction

Servo: It is a set of automatic control system composed of DC motor, transmission parts, sensors, and control circuits, which control the output angle of the rotating shaft by sending specific signals. The steering gear generally has a maximum rotation angle. With the progress of the times, electronic packaging devices have increasingly higher requirements for their performance. The substantial improvement in performance has caused a rapid increase in the internal temperature of the packaged devices. Therefore, the thermal, electrical, and mechanical performance requirements for the board-level solder joints in electronic products are also rising. According to Moore's Law proposed by Dr. Moore, every 18 months, the integration density of semiconductor crystals on active integrated circuits doubles. The control circuit is the control core of the package structure. The dense integration of the control circuit greatly increases the heat generation per unit volume of the heating device such as the chip, and generates more heat. At the same time, the number of solder joints used for the connection of the chip and the circuit board is also increasing. The solder joints in electronic packaging devices are very important in the working circuit [1], and are mostly used for mechanical support and electrical connections. Moreover, studies have shown that the high reliability of the solder joints on the control circuit board usually determines the durability of electronic packaging devices and the working life of the equipment.

## 2. Related Work

Ye et al. [2] used the GaN (gallium nitride) power amplifier chip as the basic model to analyze the thermal resistance characteristics of its heat transfer path and studied the effects of box material, thermal insulation layer material, and chip size parameters on the heat transfer path. The influence of thermal resistance provides a feasible reference solution for the heat dissipation of power amplifier chips in the future. Once the thermal resistance of the heat transfer path is studied, a feasible reference scheme is given for the heat dissipation of future power amplifier chips. Zhong et al. [3] used high-sensitivity real-time Moiré interferometry to study the interface behavior of flip-chip structures under thermal test conditions. Su et al. [4] taking the chip packaging structure as a model, using infrared thermal imaging technology, studied the change law of the chip thermal stress with the change of current, and it is found that the chip thermal stress changes logarithmically with the change of the working current. At the same time, the relationship between chip thermal stress and operating current was simulated by finite element method to verify its experimental conclusions. The thermal characteristics analysis of the subrack-level electronic packaging structure has achieved certain results under the research of domestic and foreign scholars. Liu et al. [5] studied the structural characteristics, working principle, and heat transfer process of the railway passenger car water purification tank, and established the three-dimensional finite element model of the clean water tank, and its thermal insulation process and heating process are analyzed by transient thermal simulation. The response results are compared with the experimental data to verify the reliability of the model and the research process. Mei et al. [6] used a heavy-duty gearbox as a research model. Aiming at the thermal deformation caused by the thermal load, the thermal characteristics of the gearbox structure were simulated, and the steady-state temperature field distribution of the structure was obtained. The distribution is used as the thermal load, and the response results such as stress, strain, and thermal deformation under the combined action of the thermal load and the structural load are calculated. Qi et al. [7] carried out a three-dimensional finite element modeling of the gear box, combined with the basic theory of heat transfer and finite element analysis theory to conduct a thermal-structural coupling analysis of the gear box, and obtained the temperature field distribution and heat of the gear box. According to the response results, such as deformation, the locations of the maximum stress and the maximum displacement are determined, and the factors affecting the temperature field and the deformation field are analyzed. Xu et al. [8] established a three-dimensional finite element model of a new type of ATCA subrack, and studied the heat dissipation of ATCA chips by changing the structural parameters. On this basis, they studied the influence of air-cooled heat dissipation and fan type on the heat dissipation of the structural box. The finite element simulation of ATCA sub-box structure provides suggestions for the optimal design of the structure. Yoon et al. [9]carried out a three-dimensional finite element modeling of the box structure of a railway bridge, a three-dimensional solid element meshing of the overall structure of the box, and a thermodynamic simulation by applying boundary conditions. Yang et al. [10] used Pro/E three-dimensional software for solid modeling, combined with the basic theory of thermodynamics and finite element analysis methods, and performed thermal-mechanical simulation calculations through finite element software ANSYS, and analyzed the results of its thermal response. The weak links of the box body are removed, and then a theoretical basis is provided for the optimal design of the box body structure. Huo et al. [11] conducted a thermal simulation analysis on the overall structure of the reducer, obtained the overall temperature field distribution of the reducer, and then used the temperature field distribution as the combination of the external thermal load and the static load on the reducer box for thermal-mechanical simulation analysis, Get the stress, strain, and thermal deformation distribution. This paper establishes an overall three-dimensional finite element model that integrates the steering gear control circuit board and the ball screw of the transmission component in a box with a heat insulation layer, calculates the heat source heat of each part of the ball screw of the transmission component, and explores the effect of its heat on the steering gear. The influence of the control circuit, simulating the harsh environment in which the steering gear is in service, placing the two box models with or without heat insulation layer under gradual temperature load for simulation, and comparing and analyzing whether there is heat insulation layer respond in order to explore the effect of the thermal insulation layer on the protection of the internal control circuit of the box. Ming Che et al. [12] used finite element analysis software to simulate the influence of silicon gasket on the heat dissipation of the chip in the package structure. The analysis of the calculation results shows that the better the thermal conductivity of the silicon material gasket, the more heat is transferred, and the purpose of reducing the temperature of the package structure can be achieved. Shuai and Li [13] studied the factors that cause the chip to overheat and the commonly used heat dissipation methods. At the same time, the heating plate is used as a constant heat source to simulate the heating of the computer CPU. The heat dissipation effects of five different types of fans are compared under the various power conditions of the heating plate. Wang and Shen [14] used a multi-chip double-sided PCB board as a model, and carried out finite element simulation by changing the thickness of the PCB board substrate, the size parameters of the chip, and the value of the chip power. The analysis found that the above factors all have a large effect on the temperature value.

## 3. Methods

In the text, the literature on the subject is comprehensively summarized by the method of literature research, and the key literature is refined and summarized, and then the simulation and reproduction of the same model and the same working conditions are carried out through COMSOL, and then the reproduced results are compared with the results in the literature. Carry out comparative analysis, such as simulation cloud map trends, numerical values, and curve changes. Through the comparison of the results, the error difference is small, which verifies the feasibility of this correlation method. Then, this simulation analysis method is applied to the existing project. At the same time, some data of the existing project are compared and verified by simulation and numerical value to verify the correctness of the relevant numerical value and simulation results.

## 4. The Influence of the Ball Screw of the Steering Gear on the Thermal Characteristics of the Box-Level Control Circuit

**Figure 1:**
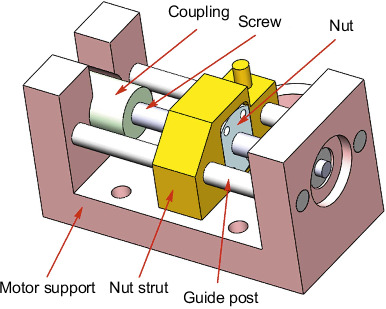
Three-dimensional structure of ball screw.

When the steering gear ball screw is in operation, the power loss of its driving motor, the bearing, and a certain amount of heat are generated due to friction, and the heat generated will affect the steering gear control circuit in the sealed steering gear device. Therefore, it is necessary to study the thermal response of the heat generated by the ball screw under the control circuit, and analyze the thermal response of the steering gear control circuit alone to explain the magnitude of the heat effect of the ball screw.  Figure 1 shows the three-dimensional structure of the ball screw.

### 4.1. Calculation of Heat Source of Steering Gear Ball Screw

#### 4.1.1. Calculate the Heat Generated by the Motor

The motor and the lead screw are connected by a coupling, so the heat of the motor is transferred to the lead screw end by heat conduction. The heat is mainly related to the output torque of the motor and the mechanical efficiency of the motor. The heating of the motor is calculated by the following formula:

(1) $$\begin{array}{c} \frac{Q=M_{T}\cdot n_{1}}{9550\left(1-\eta \right)}, \end{array}$$

formula: *Q*—heat dissipated, the unit is kW; *M*_*T*_—the output torque of the motor, the unit is *N* · *m*; *n*_1_—motor speed, the unit is r/mim; *η*—mechanical efficiency of the motor, generally 0.85–0.9, here it is 0.9.

#### 4.1.2. Calculation of Bearing Friction and Heat Generation

The friction of rolling bearings is mainly due to the heat generated by the total resistance of the components inside the bearing to the movement when the inner and outer rings of the bearing rotate. Therefore, this part of the heat is not only related to the friction torque of the bearing but also proportional to the rotational speed of the ball screw. The heat can be calculated by the following formula:

(2) $$\begin{array}{c} Q=1.047\times 10^{-4}n_{2}M, \end{array}$$

formula: *M*—frictional moment of rolling bearing; *n*_2_—screw speed.

The friction torque *M* of a rolling bearing is mainly composed of two parts, the torque *M*_0_ related to the bearing speed, type, and lubricant properties, and the friction torque *M*_1_ related to the bearing load, *M*=*M*_0_+*M*_1_. *M*_0_ is related to the bearing speed, type, and lubricant properties, and reflects the hydrodynamic loss of the lubricant.

#### 4.1.3. Ball Screw Nut Pair Calorific Value Calculation

During the operation of the ball screw, the screw rotates and drives the balls to roll along the spiral raceway. At the same time, the balls themselves are also rotating. At this time, the friction between the ball screw and the nut is expected to produce friction between the balls. The heat is the heat generated by the ball screw nut.

The calculation expression of this part of the heat is similar to the friction heat of the rolling bearing, but the friction torque of the ball screw nut pair is composed of the driving torque *M*_*D*_ and the resistance *M*_*P*_. The driving torque *M*_*D*_ of the screw refers to the driving torque that the screw drives the nut to do reciprocating motion against the axial load, Which is calculated as follows:

(3) $$\begin{array}{c} \frac{M_{D}=F_{a}P_{h}}{2\pi \eta }, \end{array}$$

formula: *F*_*a*_—axial force of the screw; *P*_*h*_—lead screw; *η*—the working efficiency of the screw nut transmission.

The resistance torque *M*_*P*_ of the ball screw refers to the preload of the screw nut pair, and the calculation formula is as follows:

(4) $$\begin{array}{c} \frac{M_{p}=F_{p}P_{h}*\left(1-\eta^{2}\right)}{2\pi }, \end{array}$$

where *F*_*P*_ is the axial preload of the screw.

By querying the above parameter data and calculating by formula, it can be obtained that the heating power of the driving motor is 6.713 W, the friction heating power of the nut pair is 5.867 W, and the friction heating power of the bearing is 0.3995 W. According to the analysis of the actual situation, the heat generated by the friction of the nut pair and the bearing is mostly diffused by internal heat conduction loss, and the heat exchanged by convection through the air medium is very small, so the heat of the ball screw is equivalent to the heat of the driving motor.

### 4.2. Analysis of the Influence of the Ball Screw of the Steering Gear on the Thermal Simulation of the Control Circuit

**Figure 2:**
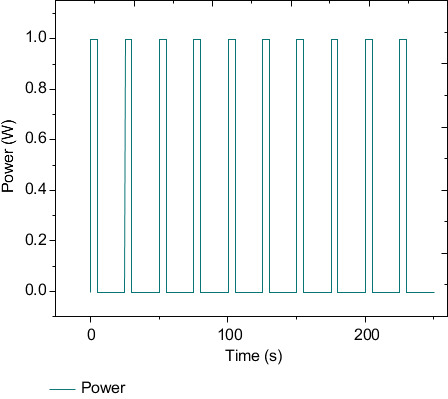
Power cycle load.

The working power of the heating source of the steering gear ball screw motor is 6.7 W, and the working model of the driving motor and the steering gear control circuit are the same. The trend is shown in Figure 2, and the motor power value is 6.7 w.

**Figure 3:**
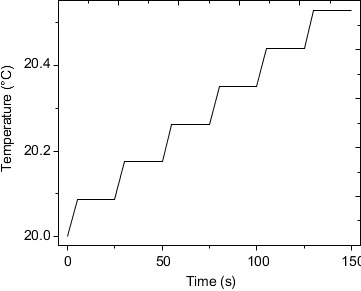
Trend chart of maximum temperature distribution of drive motor.

The temperature trend is obtained by iteratively calculating the power load applied to the drive motor by finite element.  Figure 3 shows the maximum temperature distribution trend chart of the drive motor after the end of the working cycle. The maximum temperature increases linearly during the working time and during the cooling time. Due to the large size of the motor, it is not enough to have a significant temperature change during the cooling time of this cycle. Therefore, the maximum temperature of the driving motor progresses with the cycle, and the maximum temperature shows a stepwise increase trend, and reaches the maximum temperature at the final time 20.522°C. The temperature rise rate is only 0.00348°C/s, so it is quantitatively considered that the heat source of the ball screw has little influence on the steering gear control circuit.

## 5. Establishment of a Three-Dimensional Finite Element Model of the Subrack-Level Steering Gear Control Circuit Board

### 5.1. Finite Element Model

**Figure 4:**
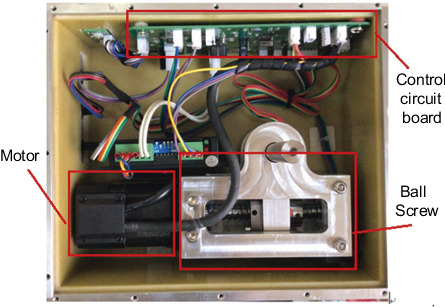
The physical diagram of the control circuit of the box-level steering gear.

**Figure 5:**
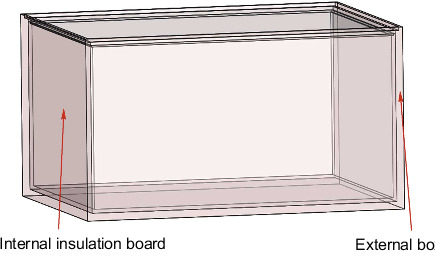
Steering gear box with heat insulation layer.

**Figure 6:**
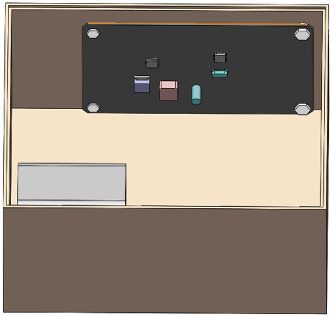
Simplified assembly drawing of the steering gear.

The physical diagram of the steering gear device is shown in Figure 4. The control circuit and the ball screw are separated on both sides and fixed with the contact surface by bolts.  Figure 5 shows the steering gear box with a 2 mm heat insulation layer designed according to the physical model. It fits seamlessly with all sides of the steering gear box to form a closed body. In order to improve the calculation efficiency, the steering gear control circuit model will be further simplified, and the SOP package with less impact on the overall body and other components far away from the heat source are ignored; in the previous section, by calculating and analyzing the heat generated by the motor, the frictional heat of the ball screw, and the heat generated by the nut pair, it is known that the frictional heat of the ball screw is mainly internal solid conduction. The influence of convection heat transfer on the control circuit is small, so in order to reduce the calculation load, the ball screw is ignored in the finite element simulation analysis model in this article. The final three-dimensional finite element model of the steering gear structure is shown in Figure 6.

### 5.2. Material Properties

In the simulation analysis of this article, the steering gear control circuit and the steering gear ball screw are assembled in the box as the research body. At the same time, the thermal characteristics of the steering gear control circuit are explored to identify whether there is a heat insulation layer and the influence of the parameters of the heat insulation layer on the thermal characteristics of the steering gear control circuit. Material is structural steel (steel structure), an epoxy material is an insulating layer, and each of the parameter values as detailed in Tables 1 and 2.

### 5.3. Meshing

**Figure 7:**
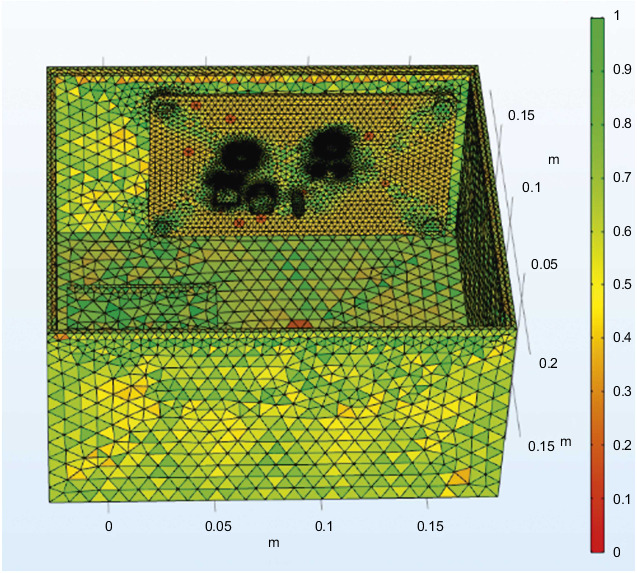
Overall finite element mesh division of the steering gear.

The whole steering gear device is mainly divided into a free tetrahedron, and the parameters of each part are customized to achieve the purpose of optimal grid division. Set the minimum unit parameter of the important component QFP package, the value is 0.001 mm, the minimum unit parameter of the chip power inductor is set to 0.01 mm, and the minimum unit parameters of the other PCB, steering gear, and heat insulation layer are set to the 0.1–5 mm range, the final overall meshing diagram is shown in Figure 7. Combined with the grid distribution diagram, it is found that the grid value around the important components is close to 1, and the quality is good. Therefore, it is believed that the grid division meets the accuracy requirements and can be used in finite element simulation calculations.

If the grid is further refined to make the grid quality reach about 0.85, the temperature inside the box is 70°C when it is stable, and the maximum temperature difference between the two is 1.4°C, it is considered that the difference is small. Within the acceptable range, it is quantitatively considered that the grid division with unit mass of 0.7955 has met the requirements of grid independence.

### 5.4. Boundary Conditions

**Figure 8:**
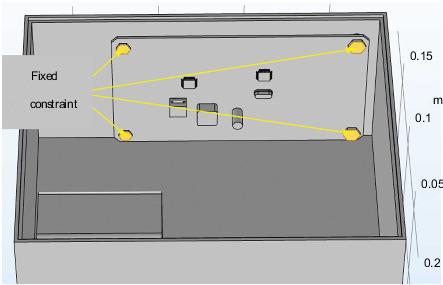
Fixed constraint setting.

The research and analysis process in the steering gear box is based on the working state of the rudder control circuit itself, so its boundary conditions are set as follows:

1. Apply 1 W cyclic power to the heating source of the steering gear control circuit. Its changing trend is shown in Figure 2. In the power cycle load, the ambient temperature is set to 20°C, the power is increased to the rated power after 0.1 s from the beginning of the cycle, and the duration is 5 s. After that, the power is reduced from the rated power to 0 after 0.1 s, and the cooling time of 20 s is a complete cycle. A full cycle time is 25 s with a 20% duty cycle. The chip loading power is 1 W. In this section, the cycle power is loaded for a total of 150 s;
2. The ambient temperature of the working condition is set to 20°C.
3. Fixing constraints are imposed on the lower surface of the steering gear box and the four bolts participating in the fastening connection, and the fixing constraints are shown in Figure 8.
4. Set the convective heat transfer coefficient on the inner and outer surfaces of the box, and the coefficient is set to 10 W/(m^2^·K).

## 6. Analysis of the Thermal Characteristics of the Thermal Insulation Layer to the Plug-In Box Level Control Circuit

### 6.1. Temperature Response Analysis

In the process of service, the steering gear will usually experience a harsher natural environment, which will have a certain impact on its internal structure. In severe cases, it will cause the steering gear to malfunction. Therefore, the existence of a thermal insulation layer should be considered at the beginning of the steering gear design. Reduce the probability of failure of the steering gear.

**Figure 9:**
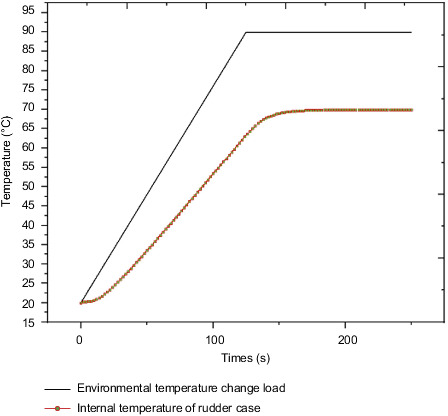
Internal and external temperature curve of steering gear.

In the finite element simulation calculation, the environmental temperature change load is applied to the outer surface of the steering gear. The environmental temperature was raised from the initial temperature of 20°C to 90°C over 125 s, and was maintained until 250 s, with a temperature rise rate of 0.5°C. The case surface is in contact with the insulation layer. The external heat is transferred from the case to the insulation layer through heat conduction, and finally the temperature of the inner surface of the heat insulation layer is measured. As the air flow rate inside the steering gear box is extremely low, the inner surface of the heat insulation layer is considered to be equivalent to the temperature inside the steering gear box. The measured temperature inside the steering gear box is shown in Figure 9. It shows that the temperature inside the box reaches a stable temperature of 68.6°C, and the heat insulation efficiency reaches 24%.

**Figure 10:**
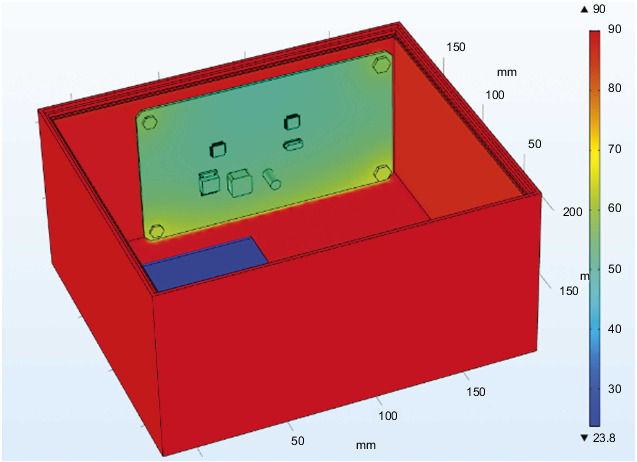
Cloud diagram of the temperature distribution of the steering gear at the final moment under the action of the heat insulation layer.

The external temperature-varying load is applied to the outer surface of the steering gear, and the measured time-varying temperature inside the steering gear is equivalent to the convective heat exchange external temperature, which is used to simulate the process of air transferring heat to the control circuit through convective heat exchange. After iterative calculation, the final temperature distribution of the steering gear under the action of the heat insulation layer is shown in Figure 10. The maximum temperature appears at the contact point between the lower surface of the control circuit and the heat insulation layer. The maximum temperature value is the ambient temperature of 90°C, and the minimum temperature value of 23.8°C appears on the drive motor. The drive motor is made of copper and has a large volume. At the final moment, there has not been a sufficiently high temperature rise, and the temperature value is low.

**Figure 11:**
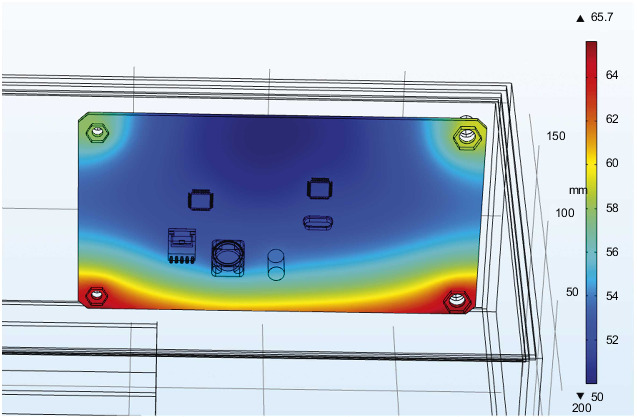
The temperature distribution cloud diagram of the PCB board at the final moment under the action of the heat insulation layer.

The steering gear control circuit is the core control component of the overall steering gear.  Figure 11 is the cloud map of the temperature distribution of the PCB board at the final moment under the action of the thermal insulation layer. The main heat sources of the PCB include three parts: including the heat transferred through the convective heat transfer of the air medium. The bolt is in direct contact with the steering gear box and the control circuit through the heat transferred by heat conduction, and the heat generated by the intermittent operation of the heating source on the control circuit is transferred to the PCB through the pin solder foot heat. Therefore, it can be found in the figure that the maximum temperature of the control circuit appears at the bottom right corner where the lower surface is in contact with the heat insulation layer and reaches 65.7°C, and the minimum value appears on the upper surface of the control circuit, and its value is 50°C. The lower surface temperature of the control circuit is higher than the others. Components and the four bolt holes are relatively high due to the presence of heat conduction. The heat generated by the chip and the patch power inductor is relatively small compared to the outside temperature, so there will be no obvious temperature gradient near it.

**Figure 12:**
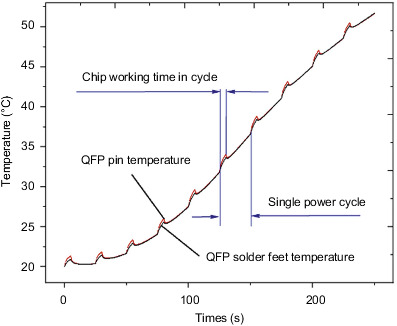
QFP package body pin and solder foot temperature change trend.

Figure 12 shows a QFP package pin and a temperature change of the fillet. The curve is derived from the QFP package with increasing time. The temperature is gradually increased. At the time, the final temperature reached 51°C. At the same time, small periodic bulges will appear during the overall temperature rise of QFP package. This is because during this period of time the chip generates a certain amount of heat due to its own power, which causes a sudden temperature rise in the corresponding period of time, resulting in bumps. In addition, the heat generated by the power of the chip is transferred from the chip to the pins and then to the solder feet. The transfer process causes a certain temperature difference between the solder feet and the pins. Therefore, the temperature of the pins is slightly greater than the temperature of the solder feet during the relevant cycle period. Since the initial temperature value of QFP is low, the temperature rise rate is lower in the early stage of the environmental temperature effect, and the curve climbing trend is slower. In the later stage of the effect, the temperature rise rate is faster and the curve climbing trend is steeper.

### 6.2. Structural Response Analysis

**Figure 13:**
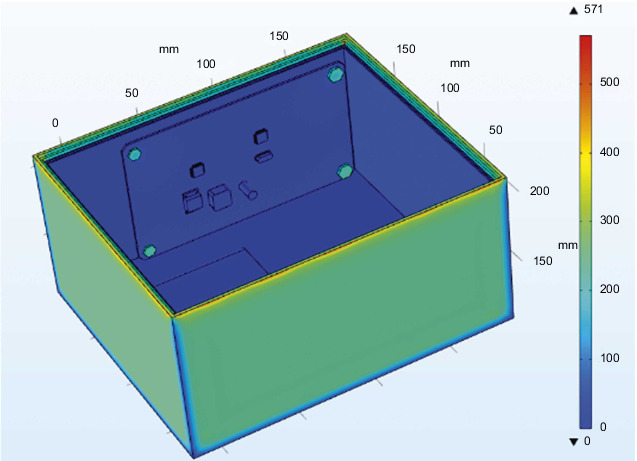
The stress distribution cloud diagram of the steering gear at the final moment under the action of the heat insulation layer.

Import the above temperature results into the structure module to calculate the structure results, as shown in Figure 13, which is a cloud diagram of the stress distribution of the steering gear at the final moment under the action of the heat insulation layer. Because the ambient temperature of the steering gear box and the steering gear itself has a larger temperature gradient, the wall of the steering gear produces a larger stress value, and the maximum stress value is at the edge position where the cover plate contacts the case body, which is a stress concentration phenomenon.

**Figure 14:**
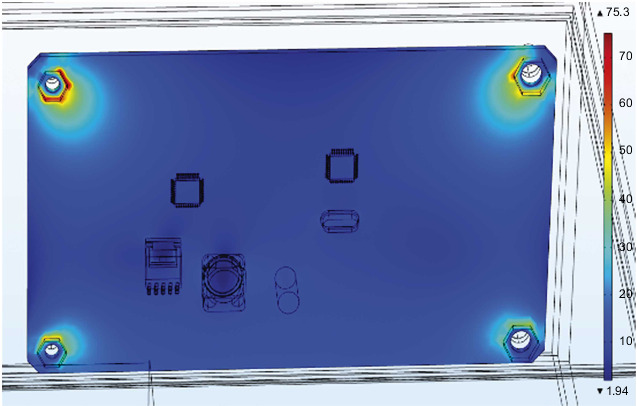
The stress distribution cloud diagram of the PCB board.

**Figure 15:**
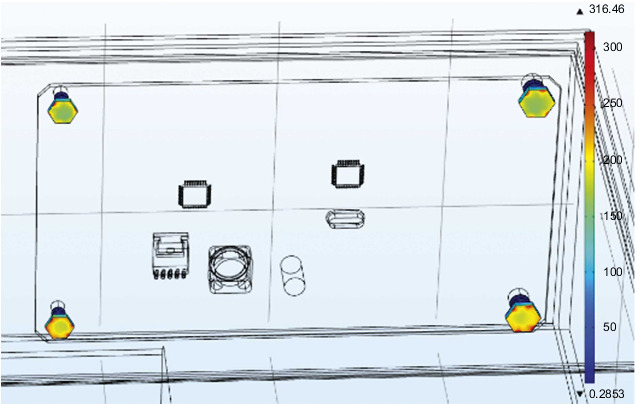
Stress distribution cloud diagram of four bolts.

Since the overall stress gradient distribution of the steering gear is quite different, the PCB and the four bolts are separately analyzed for stress. As shown in Figure 14 is the stress distribution cloud diagram of the PCB board, the maximum stress appears on the surface of the bolt hole in the upper left corner, and the value is 75.3 MPa; Figure 15 is a cloud diagram of the stress distribution of four bolts. The maximum stress appears on the surface of the bolt at the lower right corner, and its value is 316 MPa. Query data learned by, the PCB yield strength of 350 MPa, yield limit of the steel of 454 MPa, the PCB and the bolt stress values are less than the corresponding yield limit.

**Figure 16:**
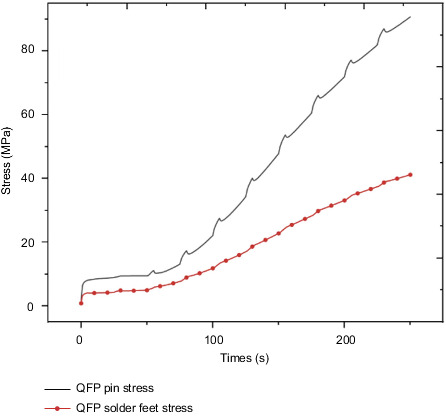
QFP package body lead and solder foot stress trend.

Figure 16 shows the stress distribution trend diagram of the first group of pins and solder feet of the QFP package. The change trend is similar to the temperature change trend, and the stress value gradually increases with time. Among them, the copper pin stress change trend has obvious bump changes with the change of the chip power cycle, and the tin-lead soldering pin changes smoothly with the cycle. At the final moment, the stress value of the copper pin reached 90.7 MPa, and the stress value of the tin-lead solder pin reached 41 MPa.

Through the analysis of the stress value of PCB, four bolts and QFP pin solder foot, combined with the corresponding yield limit of each material and their respective common failure modes, it is conservatively considered that the common failure location of the steering gear control circuit is QFP under severe environmental conditions—the connection point between the pin and the solder foot.

## 7. Analysis of Thermal Characteristics of Sub-Box-Level Control Circuit with or without Heat Insulation Layer

**Figure 17:**
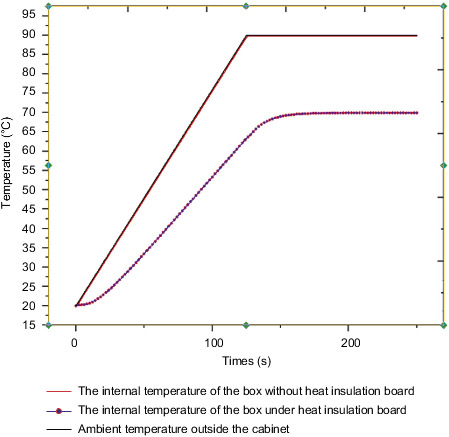
The internal temperature curve of the box with or without the heat insulation layer.

In the previous section, the thermal response results of the steering gear box and the internal control circuit under the action of the heat insulation layer were elaborated. This section quantitatively analyzes the influence of thermal insulation by comparing the thermal characteristic response under the action of thermal insulation. The lower surface of the internal control circuit of the thermal insulation model is in contact with the thermal insulation, while the model without the thermal insulation is in contact with the surface of the box. In the finite element analysis and calculation, Figure 17 shows the internal temperature curve of the cabinet with or without the heat insulation layer. From the figure, it can be seen that the internal temperature value without the heat insulation layer is close to the external ambient temperature. When it reaches 89.8°C, the internal temperature value under the action of the heat insulation layer is 68.6°C. Only from the internal temperature performance analysis of the box, the thermal insulation efficiency of the thermal insulation layer is 24%, and the thermal insulation effect is significant.

**Figure 18:**
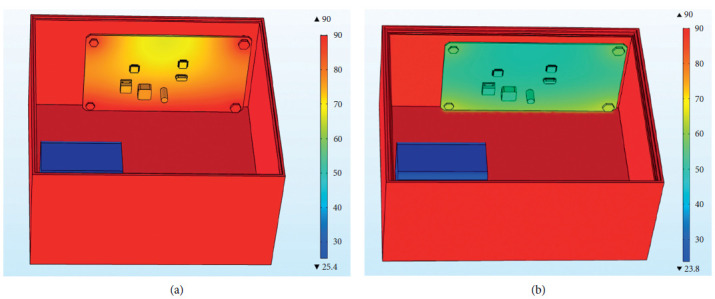
Cloud diagram of overall temperature distribution with or without heat insulation layer. (a) Cloud diagram of overall temperature distribution without heat insulation layer. (b) Cloud map of the overall temperature distribution under the action of the thermal insulation layer.

**Figure 19:**
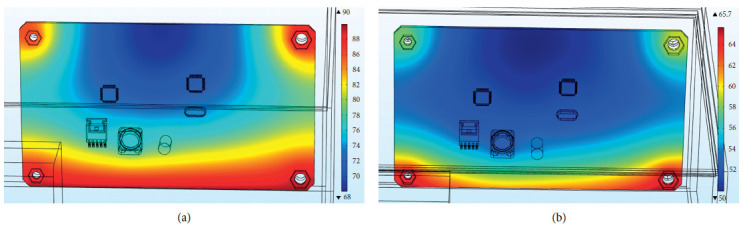
PCB board temperature distribution with or without heat insulation layer. (a) PCB board temperature distribution without heat insulation layer. (b) PCB board temperature distribution under the action of the heat insulation layer.

The external temperature change load is applied to the outer surface of the steering gear, and the measured time-varying temperature inside the steering gear is equivalent to the external temperature of convective heat transfer.  Figure 18 shows the overall temperature distribution cloud diagram with or without heat insulation. In the figure, it can be seen that the overall temperature of the internal control circuit without the effect of the heat insulation layer is significantly higher than that of the internal control circuit under the effect of the heat insulation layer. The two models have similar temperature distribution trends on the control circuit. The temperature of the four bolts is higher than that of other parts of the control circuit board, and the temperature of the middle position is lower. As shown in Figure 19, the temperature distribution of PCB with or without insulation layer is completely consistent. The maximum temperature of PCB without insulation layer is 90°C, and the minimum temperature is 68°C. The average temperature of the PCB board is 79°C; the highest temperature of the PCB board under the action of the heat insulation layer is 65.7°C, the minimum temperature value is 50 °C, and the average temperature of the PCB board is 57.4°C. The overall temperature of the two models is quite different.

**Figure 20:**
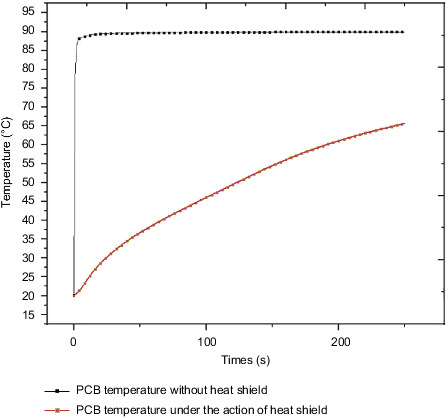
PCB board temperature with or without the heat insulation layer.

Figure 20 shows the maximum temperature curve of the PCB board with or without the heat insulation layer. In the model without the heat insulation layer, since the lower surface of the control circuit and the bolt surface are directly in contact with the steering gear box, the structural steel material has good thermal conductivity; therefore, the temperature rise rate of the PCB board is very fast, reaching the maximum value of 89.2 °C in a short time, and the temperature change curve is steep; in the insulation layer model, due to the existence of the insulation layer, the temperature that reaches the inside within the same time is much less than where there is no insulation layer. In the thermal layer model, the maximum temperature at the final moment is 65.7 °C, and from the figure, it can be concluded that the temperature growth trend of the PCB board is relatively flat.

**Figure 21:**
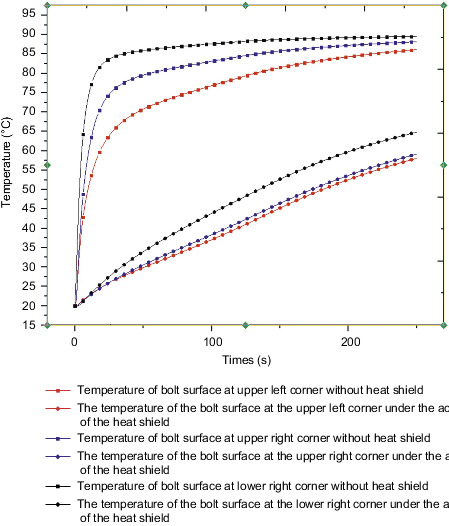
Bolt surface temperature with or without the heat insulation layer.

Figure 21 shows the temperature curves of the three bolt faces with or without the heat insulation layer. The analysis shows that the temperature values of the left and right bolts on the lower surface are similar. Here, we only study the temperature of the three bolt faces. The analysis shows that there is no heat insulation. The bolt temperature value and the temperature rise rate in the layer model are greater than those in the insulation layer model. A comparative analysis of the maximum temperature changes of PCB boards and bolts in the model with or without the insulation layer is carried out. The temperature and rate of temperature rise of the insulation layer model are lower than those of the model without insulation layer, and the values are quite different.

**Figure 22:**
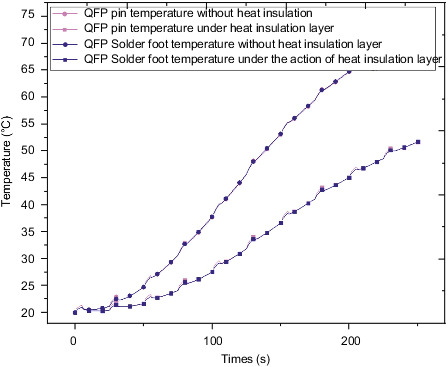
QFP pin solder foot temperature with or without heat insulation layer.

Figure 22 shows the temperature curve of QFP pin solder fillet with and without thermal insulation layer. The temperature value of QFP pin solder pin under the thermal insulation layer model and the QFP pin solder pin under the model without thermal insulation layer are almost the same. At the same time, it can be found that the temperature values at the same location in the two models are quite different.

**Figure 23:**
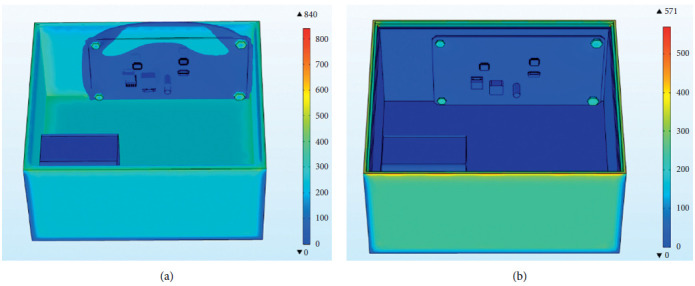
Overall, stress cloud diagram with or without heat insulation layer. (a) Overall stress cloud diagram without heat insulation layer. (b) The overall stress cloud diagram under the action of the insulation layer.

Figure 23 shows the overall stress cloud diagram with or without the thermal insulation layer. It can be concluded that stress concentration occurs in the overall stress distribution with or without the thermal insulation layer. Among them, the overall stress without the thermal insulation layer is greater than that with the thermal insulation layer. With the overall stress under the action, the maximum stress on the control circuit board inside the box still appears on the bolt surface.

**Figure 24:**
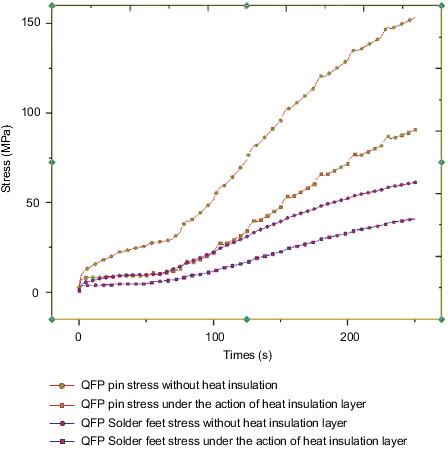
QFP pin solder foot stress with or without heat insulation layer.

The QFP pin soldering foot of the dangerous component on the control circuit is selected as the research object.  Figure 24 shows the stress of the QFP pin soldering foot with or without the heat insulation layer. The final stress values of the pins without the heat insulation layer are 153 MPa and 90 MPa, respectively; the final stress values of the pins under the action of the heat insulation layer are 61 MPa and 41 MPa, respectively. The solder foot is made of tin-lead material, and the required stress value is 45 MPa. Therefore, the solder foot failure phenomenon may occur without the action of the heat insulation layer, which will cause the steering gear to fall into failure mode. By comparing the temperature and stress of the PCB board, bolts, and QFP pin solder feet under the action of the heat insulation layer, the values of the two are quite different, and the effect of the heat insulation layer is significant. Therefore, it is considered that heat insulation should be considered at the beginning of the design. Layers are necessary and indispensable.

Through comparative analysis of the temperature and stress response results of PCB board, bolts, and QFP pin solder feet under the action of the heat insulation layer, the response value under the action of the heat insulation layer is much smaller than the corresponding value under the action where there is no heat insulation layer In response to the numerical value, the effect of the insulation layer is significant, so it is considered necessary to consider the insulation layer at the beginning of the design.

## 8. Comparative Analysis of Thermal Characteristics of Different Parameters of Insulation Layer

### 8.1. Comparative Analysis of Thermal Properties of Different Thermal Conductivities of Thermal Insulation Layers

The thermal insulation layer of different materials has different thermal conductivity, and the heat transfer from the external environment to the inside through the heat conduction method is different, which makes the temperature gradient generated by the internal heat acting on the control circuit different. This section aims to study the thermal response of different materials and selects the one with better comprehensive performance of the selected materials by comparative analysis.

This section selects foamed polyurethane (EPU) materials with a thermal conductivity of 0.047 and asbestos materials with a thermal conductivity of 0.152, and comprehensively compares and analyzes the epoxy resin materials with a thermal conductivity of 0.1 studied in the previous section, from the aspects of thermal insulation effect and economic cost. Consider choosing a more suitable insulation material.

**Figure 25:**
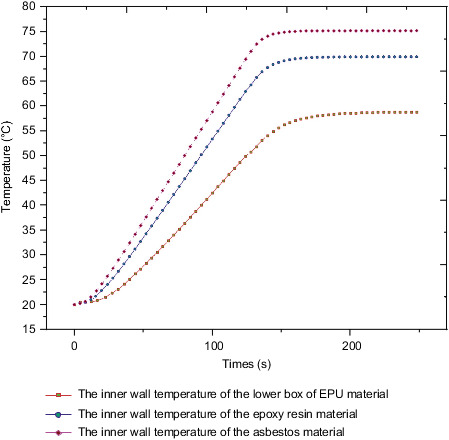
The internal temperature of the box under different materials.

Figure 25 shows the internal surface temperature curve measured under the action of different insulation layers when the external environmental load is applied to the box body. The internal temperature reached is also different due to the different thermal conductivity of each material. Under the action of EPU, epoxy resin, and asbestos materials, the internal temperature reaches 58.8, 69.9, and 75.1, respectively. Due to the certain hysteresis of heat conduction, the time node when the internal temperature reaches stability under the action of the three thermal insulation materials is greater than the time node when the ambient temperature reaches stability.

**Figure 26:**
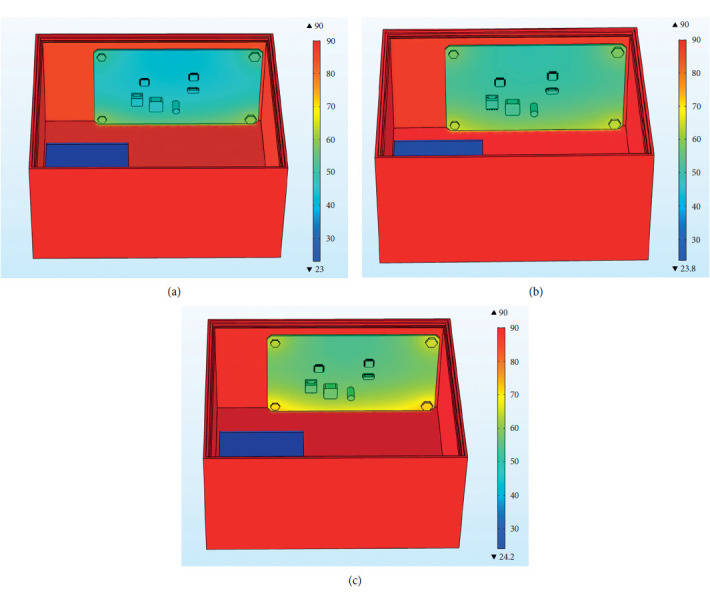
Overall temperature cloud diagram under different materials. (a) Overall temperature cloud diagram under EPU material. (b) Overall temperature cloud diagram under epoxy resin material. (c) Overall temperature cloud diagram under asbestos material.

Figures 26(a)–26(c) are the overall temperature cloud diagrams under EPU, epoxy resin, and asbestos materials, respectively. It can be found from the figure that the overall temperature of the internal control circuit under the action of the EPU heat insulation layer material is the lowest, and in the asbestos the overall temperature of the internal control circuit under the action of the heat insulation layer material is the highest. With the increase of the thermal conductivity, the temperature of the internal control circuit increases. The temperature trend under the action of the three materials is the same, and the temperature value of the four bolts is higher than that of other positions of the PCB.

**Figure 27:**
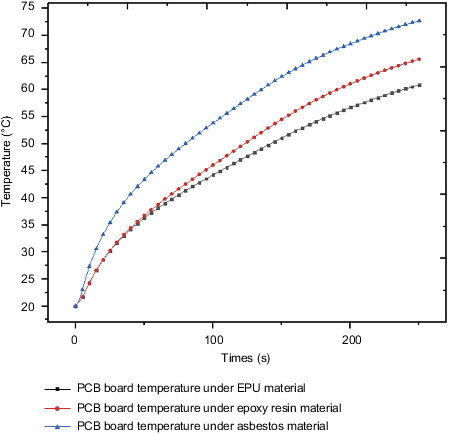
PCB board temperature under the action of different insulation materials.

**Figure 28:**
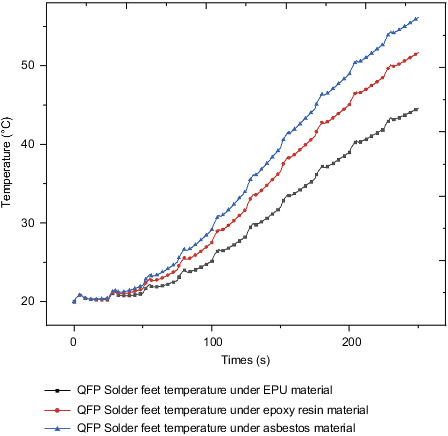
Welding foot temperature under the action of different insulation materials.

The control circuit is located in the steering gear and exchanges heat with internal heat through convection heat exchange. The temperature rise trend of different components on the control circuit is analyzed separately, and PCB and QFP solder feet are selected to study the temperature rise trend.  Figure 27 shows the temperature rise curve of the PCB board under the action of three different heat insulation materials. The corresponding materials are EPU, epoxy resin, and asbestos. The maximum temperature of the PCB board at the final moment is 60.9°C, 65.6°C, and 72.7°C. The maximum temperature increase was 7.8% and 10.8% in sequence.  Figure 28 shows the temperature rise curve of QFP soldering feet under the action of different insulation materials, corresponding to EPU, epoxy resin, and asbestos. The maximum temperature of QFP soldering feet at the final moment is 44.6°C, 51.7°C, and 56.2°C. The maximum temperature increase was 15.9% and 8.7% in sequence. The PCB board and QFP comparative analysis of the temperature rise curve of the solder foot shows that the temperature rise curve rate of the PCB board is first large and then small, and the temperature rise rate of the QFP solder foot is first small and then large. The reason is that the temperature of the PCB increases through the heat conduction method. The main heat source of the QFP is Convection heat exchange with the internal air causing the temperature to rise.

**Figure 29:**
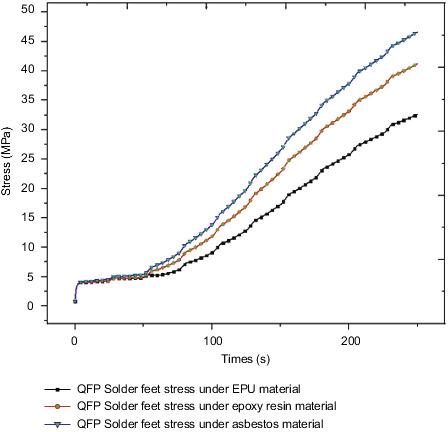
QFP welding foot stress under different heat insulation materials.

Since the overall stress cloud chart trends under the action of different insulation materials are completely consistent, the QFP solder foot with obvious stress law is selected as the research object for stress analysis.  Figure 29 shows the stress curve of QFP welding foot under the action of different insulation materials. For EPU, epoxy resin, and asbestos, the maximum stress value of QFP welding foot at the final moment is 32.6 MPa, 41.2 MPa, 46.6 MPa, respectively, and the maximum stress increase was 26.9% and 13.1% in sequence. The stress change curve of the welding foot under the action of different insulation layer materials is similar to the temperature change curve, but the stress change is more significant than the temperature change.

Through the temperature and stress comparison analysis of different components of the control circuit under the action of different insulation materials, it is found that the thermal conductivity of the EPU insulation material is the smallest compared with the other two materials, and the insulation effect is the best, but the EPU material is more expensive in the market. Under the action of asbestos as the insulating layer material, the stress value of the QFP soldering leg reaches 46.6MPa; at the final time, the soldering leg is made of tin-lead, and its allowable stress value is about 45MPa. The internal control circuit may fail under the action of the insulating layer material asbestos. Therefore, considering the heat insulation effect and economic cost, the epoxy resin material is selected as the heat insulation material for this product.

### 8.2. Comparative Analysis of Thermal Characteristics of Different Thickness Insulation Layers

For certain insulation layer materials, different insulation layer thicknesses have different thermal resistances, so that the internal temperature under the action of insulation layers with different thicknesses is not nearly the same. This section studies the insulation layer under the condition that the insulation layer material has been determined for the influence of thickness; the optimal thickness of the thermal insulation layer is also selected from various factors such as thermal insulation effect and cost. This section selects the epoxy resin insulation material determined in the previous section to study the internal control circuit under the action of three different insulation layers of thickness 2 mm, 3 mm and 4 mm, and compares and analyzes the corresponding responses of their important components and comprehensively considers various aspects. Determine the appropriate insulation thickness.

**Figure 30:**
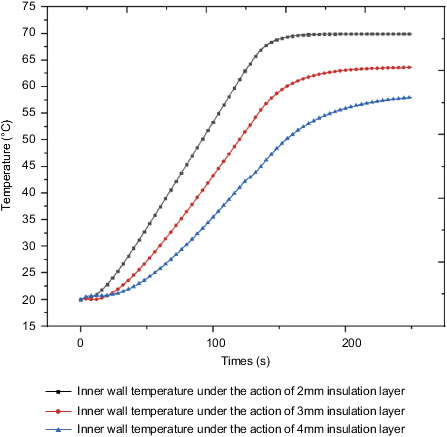
Internal temperature under the action of different thicknesses of the heat insulation layer.

Figure 30 shows the internal surface temperature curve measured under the action of different insulation layers when the external environmental load is applied to the outside of the box. As the thickness of the thermal insulation layer increases, the thermal resistance increases, and the temperature of the inner wall of the box decreases. The temperature of the inner wall of the thermal insulation layer with a thickness of 2 mm is 69.9°C at the final moment, and the temperature of the inner wall of the thermal insulation layer with a thickness of 3 mm is 63.6°C at the final moment. The inner wall temperature of the 4mm thickness insulation layer is 58.0°C at the final moment, and the inner wall temperature of the box decreases by about 9% for every 1mm increase in the thickness of the insulation layer.

**Figure 31:**
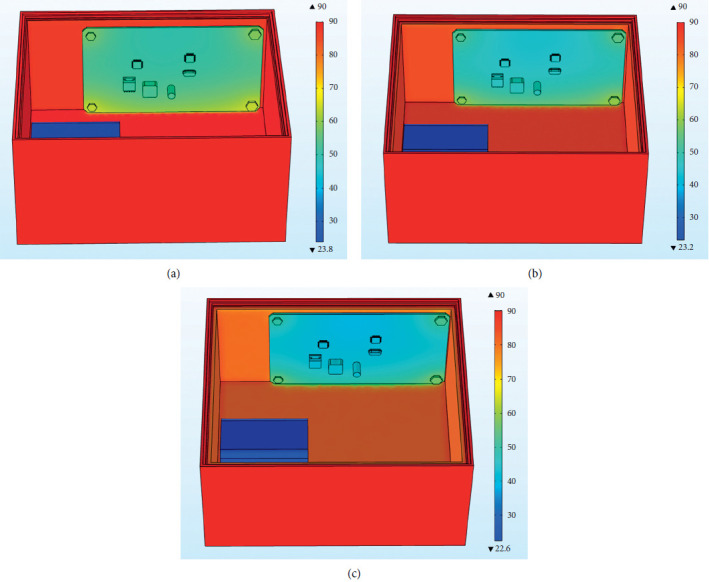
Temperature cloud diagram under the action of insulation layers of different thicknesses. (a) Temperature cloud diagram under the action of 2 mm insulation layer. (b) Temperature cloud diagram under the action of 3 mm insulation layer. (c) Temperature cloud diagram under the action of 4 mm insulation layer.

Figures 31(a)–31(c) are the overall temperature cloud diagrams under the action of 2 mm, 2 mm, and 4 mm insulation layers, respectively. As the thickness of the insulation layer increases, the temperature of the internal control circuit decreases. Increasing the thickness of the thermal insulation layer helps to reduce temperature of the control circuit and reduce the probability of failure.

**Figure 32:**
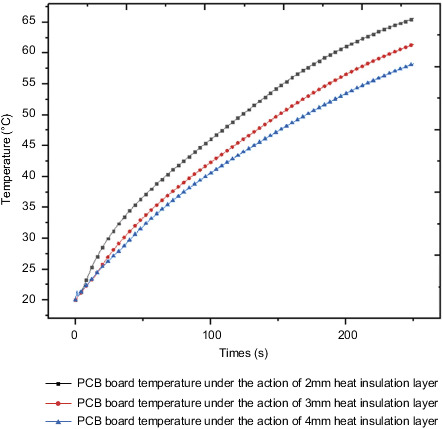
PCB board temperature under the action of different thicknesses of the heat insulation layers.

**Figure 33:**
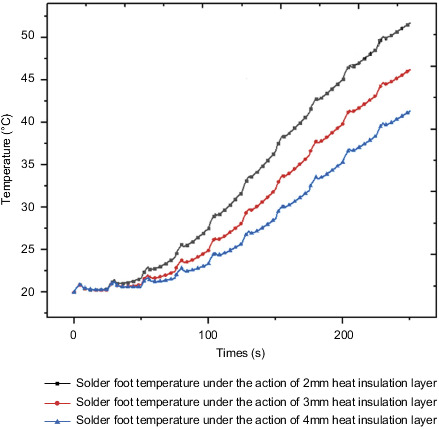
QFP solder foot temperature under the different thicknesses of the heat insulation layers.

Figure 32 shows the temperature rise curve of the PCB board under the action of three different thicknesses of the heat insulation layer. Under the action of 2 mm heat insulation layer, 3 mm heat insulation layer, and 4 mm heat insulation layer, the maximum temperature value of the PCB board at the final moment is 65.6°C, 61.5°C, and 58.3°C, with the gradual increase of the thickness of the thermal insulation layer, and the maximum temperature drop ratios are 10.6% and 10.6% in turn.  Figure 33 shows the temperature rise curve of QFP welding feet under the action of different thicknesses of heat insulation layers. Under the actions of 2 mm heat insulation layer, 3 mm heat insulation layer, and 4 mm heat insulation layer, the maximum temperature value of QFP solder feet at the final moment is 51.7°C, 46.2°C, 41.3°C, with the gradual increase of the thickness of the thermal insulation layer, and the maximum temperature drop ratios are 6.3% and 5.2% in turn.

**Figure 34:**
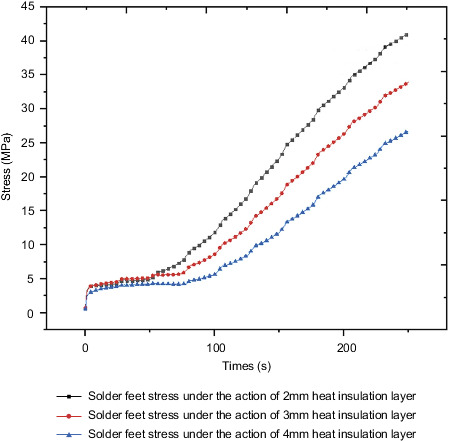
QFP welding foot stress under different thicknesses of the heat insulation layer.

Figure 34 shows the stress curve of QFP welding foot under the action of different thicknesses of the heat insulation layer. Under the action of 2 mm heat insulation layer, 3 mm heat insulation layer, and 4 mm heat insulation layer, the maximum stress value of QFP solder foot at the final moment is 41.2 MPa, at 33.9 MPa, and 26.8 MPa, with the gradual increase of the thickness of the thermal insulation layer, and the maximum stress drop ratios are 17.7% and 20.9% in turn. The influence of stress changes under the action of different thickness insulation layers is greater.

Through the comparison of the overall temperature under the action of different thicknesses of the heat insulation layer and the temperature of different components of the control circuit, it is known that as the thickness of the heat insulation layer increases, the internal temperature and heat act on the control circuit through convective heat transfer. The resulting temperature will decrease accordingly. By inquiring the electronic manual, the working permission temperature of related components on the steering gear was obtained. When the thickness of the heat insulation layer is determined to be 2 mm, the internal ambient temperature of the box has already met the working permission temperature.

Therefore, from the cost point of view, the thickness of the insulation layer of 2 mm thickness has met the working requirements; at the same time, it is known from the above analysis that the stress is more sensitive under the action of the insulation layer of different thickness, and the stress changes greatly. For high requirements, a 4-mm thick thermal insulation layer or even a larger thermal insulation layer can be used.

## 9. Conclusion

This paper establishes an overall three-dimensional finite element model that integrates the steering gear control circuit and the steering gear ball screw in the box, studies the influence of the internal ball screw heating source on the control circuit, and analyzes and calculates that the heat source of the ball screw is relatively high. The impact on the control circuit is small.

1. In order to simulate the effect of the heat insulation layer of the steering gear box in the harsh natural environment during the service process, the case body is placed at a gradual temperature and finally kept at a constant high temperature environment, and a comparative analysis is made from the three perspectives of temperature and stress. Based on the finite element response of two models with and without the insulation layer, it is concluded that the effect of the insulation layer is significant, and the insulation layer is necessary in the process of design and use;
2. Through the analysis of the thermal response of the steering gear under the conditions of different materials and different thicknesses of the heat insulation layer, the design of the heat insulation layer is further optimized and recommended.

## Figures and Tables

**Table 1:** Thermal simulation material properties.

Material	Thermal conductivity (W/(m K))	Density (kg/m^3^)	Specific heat capacity (J/(Kg K))
Structural steel	44.5	7859	475
Epoxy resin	0.1	980	1000

**Table 2:** Thermal-mechanical simulation material properties.

Material	Young's modulus (MPa )	Poisson's ratio	Thermal expansion coefficient [1/K]
Structural steel	2E5	0.3	1.23*E*-5
Epoxy resin	1.2E5	0.3	6.71*E*-5

## Data Availability

The data used to support the findings of this study are available from the corresponding author upon request.
